# Age-related hearing loss in healthy older adults is associated with arterial stiffening and higher aortic systolic blood pressure: potential role of inflammation

**DOI:** 10.3389/fragi.2026.1642659

**Published:** 2026-02-11

**Authors:** Nicholas A. Carlini, Lynn M. Bielski, Courtney N. Mudd, Taylor C. Harman-Hornbeck, Matthew P. Harber, Bradley S. Fleenor

**Affiliations:** 1 Clinical Exercise Physiology, Human Performance Laboratory, Ball State University, Muncie, IN, United States; 2 Department of Speech Pathology and Audiology, Ball State University, Muncie, IN, United States; 3 Department of Kinesiology, Taylor University, Upland, IN, United States; 4 DeBusk College of Osteopathic Medicine, Lincoln Memorial University, Harrogate, TN, United States

**Keywords:** aging, presbycusis, vascular hemodynamics, aortic stiffness, blood pressure

## Abstract

Age-related aortic stiffening increases aortic (central) blood pressure and flow pulsatility, resulting in microvascular dysfunction and target organ damage. The relationship between aortic stiffness, aortic blood pressure, and age-related hearing loss has not been fully determined. We hypothesize that aortic stiffness and aortic blood pressure will be associated with hearing loss and attenuated by inflammatory biomarkers [matrix metalloproteinase-2 (MMP-2), resistin, and vaspin]. Twenty-two younger (n = 11, 4M/7F, age 25.5 ± 2.1 years) and older (n = 11, 4M/7F, age 65.8 ± 0.9 years) adults completed resting measures of aortic stiffness (carotid–femoral pulse wave velocity, cfPWV), pulse wave analysis, and hearing sensitivity. Compared to young adults, older adults had higher cfPWV, aortic systolic blood pressure (aSBP), speech recognition thresholds (SRT), and pure tone averages (PTA) in the low (LFPTA) and high frequency (HFPTA) domains (all, p < 0.05). cfPWV was correlated with right ear (RE) LFPTA (r = 0.45, p = 0.04) and HFPTA (r = 0.53, p = 0.01) and left ear (LE) HFPTA (r = 0.52, p = 0.02). aSBP was correlated with RE SRT (r = 0.47, p = 0.03), LE SRT (r = 0.44, p = 0.04), RE LFPTA (r = 0.41, p = 0.05), and RE (r = 0.53, p = 0.01) and LE HFPTA (r = 0.46, p = 0.03). The relationship between cfPWV and aSBP and select PTA did not remain after adjusting for MMP-2 and resistin (p > 0.05). These data provide novel insights demonstrating that aortic stiffness and aSBP are related to reduced hearing sensitivity, which may, in part, be mediated by inflammation.

## 1 Introduction

Older age is a primary risk factor for greater cardiovascular disease (CVD) risk, which is attributable, in part, to stiffening of the aorta (Mitchell, 2021). Aortic stiffness, when assessed by carotid–femoral pulse wave velocity (cfPWV), is associated with higher resting blood pressure of the peripheral (brachial) and central (aortic) arteries and predicts future CVD events (Mitchell et al., 2010). As such, age-related aortic stiffening has the potential to promote excess flow pulsatility contributing to a greater hemodynamic load, resulting in microvasculature dysfunction and damage to high-flow, low-resistance organs and tissues (Chirinos et al., 2019). Specifically, dysfunction of the heart, brain, and kidneys has been associated with a greater pulsatile load due to aortic stiffening.

Age-related hearing loss, or presbycusis, is typically seen in both ears and is characterized by the loss of pure tone hearing sensitivity and the reduction of speech understanding (Lee, 2013). Presbycusis affects ∼33% of adults over the age of 65 years, contributing to frailty, depression, cognitive dysfunction and increased risk of dementia, social isolation, and loneliness, especially if left untreated (Lv et al., 2023). A key organ in the peripheral auditory system involved in the encoding of sound is the cochlea. Given the rich blood supply to the stria vascularis, a highly vascularized tissue lining the cochlea, it is reasonable to infer that factors affecting blood flow and blood pressure, such as aortic stiffness, could influence hearing sensitivity and auditory function. Furthermore, thickening of the capillary wall in the stria vascularis increases with aging, contributing to inadequate blood supply to the cochlea (Yoshioka et al., 2010; Thulasiram et al., 2022). Limited evidence supports the notion that changes in cochlear structure and function are related to microvascular changes in blood supply, with conflicting findings indicating that hearing loss and sensitivity may or may not be associated with elevated blood pressure (Gates et al., 1993; Friedland et al., 2009; Tan et al., 2018; Wattamwar et al., 2018). More recent evidence suggests that central blood pressure due to greater aortic stiffening may have a greater influence on target organ damage (McEniery et al., 2014). Although few studies (Helzner et al., 2011; Chung et al., 2016) have examined the relationship between age-related hearing loss and arterial stiffness, the augmentation index normalized to heart rate 75 (AIxHR75), central blood pressure, and the factors potentially influencing this relationship remain unknown.

A primary mechanism underlying age-related increases in aortic stiffness and blood pressure is inflammation (Sun, 2015; Fleenor et al., 2022; Murray et al., 2023). Preclinical animal and clinical human studies have identified inflammation as a pathophysiological mechanism contributing to presbycusis (Paplou et al., 2021). Specifically, systemic inflammation (e.g., C-reactive protein) in adults with hearing loss is associated with elevated hearing thresholds, indicating the loss of hearing sensitivity (Verschuur et al., 2012; Verschuur et al., 2014). Novel age-related markers of inflammation including adipocytokines such as resistin, vaspin, and matrix metalloproteinases (MMPs) may also contribute to auditory dysfunction (Manicone and McGuire, 2008; Tripathi et al., 2020; Park and Shimokawa, 2024). Previous evidence demonstrates that resistin is a primary regulator of inflammation in several vascular cell types and is associated with aortic stiffness and hypertension, whereas vaspin is anti-inflammatory (Norman et al., 2016; Zhang et al., 2017; Radzik-Zając et al., 2023). Furthermore, MMPs play a role in regulating the cochlear response to acoustic trauma and in vascular remodeling through the degradation of the extracellular matrix such as collagen and elastin, which are present in the cochlea (Filková et al., 2009; Chen et al., 2013; Wu et al., 2017; Simões et al., 2022). Collectively, these findings support the notion for inflammation as an underlying mechanism contributing to age-related hearing loss. However, it is currently unknown if the relationship between aortic stiffness, central blood pressure, and hearing loss is influenced by inflammation.

The purpose of this exploratory study was to determine if age-related increases in aortic stiffness and central blood pressure are related to hearing loss/sensitivity and examine the potential impact of inflammation on this relationship. We hypothesize that 1) aortic stiffness and central blood pressure will be correlated with poorer hearing sensitivity by measures of speech recognition threshold (SRT) and low- and high-frequency pure tone average (PTA) and 2) the relationship between aortic stiffness, central blood pressure, and hearing loss will be influenced, in part, by resistin, vaspin, and MMP-2.

## 2 Materials and methods

All procedures for this study were reviewed and approved by the Ball State University Institutional Review Board. The benefits and risks associated with the study procedures were explained to all participants prior to obtaining written informed consent before starting the study. All participants reported to the Clinical Exercise Physiology (CEP) Laboratory at Ball State University for all vascular hemodynamic assessments and measurements of aortic stiffness. All cardiovascular assessments were performed after an overnight fast (8 h–12 h) from food, exercise, caffeine, and alcohol. All audiometric measures were performed in the Speech Pathology and Audiology Clinic in the Health Professionals Building at Ball State University.

### 2.1 Participants

Twenty-two (8M/14F) apparently healthy young (n = 11, age 25.5 ± 2.1 years) and older (n = 11, age 65.8 ± 0.9 years) Caucasian adults participated in this cross-sectional proof-of-concept pilot study investigation. All participants were free of any known cardiovascular or metabolic disease and cancer. Participants with the following conditions were excluded: brachial systolic blood pressure >140 mmHg or diastolic >90 mmHg, current smoker, history of head injuries, or stroke. Participants with the following auditory characteristics were excluded: hearing loss present at birth, currently using a hearing aid or amplification device, and asymmetric pure tone thresholds. Participants in the younger group were excluded if they had pure tone hearing thresholds outside the normal range (<20 dB HL) from 500 to 8,000 Hz. Older women were post-menopausal, which was defined as ≥1 year since the last menses.

### 2.2 Resting heart rate, anthropometrics, body composition, and blood lipids

The resting heart rate was measured using a finger pulse oximeter (Masimo, Irvine, California). Anthropometrics including body mass index (BMI, kg/m^2^) and body composition including total body fat percentage were calculated from the height and weight and dual x-ray absorptiometry (iDXA, GE HealthCare) as described previously (Carlini et al., 2023). All blood lipid analyses for total cholesterol, low-density lipoprotein (LDL), high-density lipoprotein (HDL), triglycerides, fasting blood glucose, and high-sensitivity C-reactive protein (hs-CRP) were measured at a local diagnostic laboratory (Labcorp Inc. Muncie, IN).

### 2.3 Inflammatory biomarkers

All blood samples were collected in the fasted condition and stored at −80 °C until analyzed. Markers of inflammation were measured using enzyme-linked immunoassays (ELISAs). Inflammatory markers included matrix metalloproteinase-2 (MMP-2) (R&D Systems, Cat. #MMP200), resistin (R&D Systems, Cat. #DRSN00), and vaspin (R&D Systems, Cat. #DSA120). All samples were performed in duplicate.

### 2.4 Resting brachial and central vascular hemodynamics

Resting brachial blood pressure (BP) and central (aortic) vascular hemodynamics were assessed as previously described (Carlini et al., 2024a). Resting brachial BP was measured by a trained CEP staff member using a manual sphygmomanometer after the participants rested in the seated position for ∼5 min. Two BP within 6 mmHg for systolic and 4 mmHg for diastolic pressure were averaged and used for analysis. Central hemodynamics including aortic BP, pulse pressure (PP, systolic–diastolic blood pressure), augmentation index (AIx, augmentation pressure-PP*100), and AIxHR75 (calculated as [−0.48*(75 − heart rate)] + AIx) were assessed in the supine position from pulse wave analysis (PWA) using the SphygmoCor XCEL device. Brachial arterial waveforms were acquired from a brachial BP cuff inflated to sub-diastolic pressures to estimate central BP from a device-specific brachial–aortic transfer function (Shoji et al., 2017). Two acceptable measurements (brachial systolic BP ≤ 5 mmHg and AIxHR75 within 5%) that underwent quality control were averaged and used for analysis.

### 2.5 Aortic stiffness

Aortic stiffness, assessed via cfPWV, was obtained using the SphygmoCor XCEL device on the right side of the body in the supine position as previously described (Fleenor et al., 2021; Carlini et al., 2024b). Carotid applanation tonometry was performed simultaneously, while a BP cuff fitted to the top of the thigh acquired femoral pulse waves. cfPWV is presented in meters/second (m/s), where the distance between the carotid and femoral pulse waves was divided by the transit time delay. Two cfPWV measurements within 0.5 m/s were averaged and used for analysis.

### 2.6 Audiology measurements

All participants underwent audiological testing including air and bone conduction audiometry. Pure tone threshold values were obtained from 500 Hz to 8,000 Hz using EAR-3A insert earphones, an Otometrics audiometer, and a Radio Ear B-71 bone oscillator. Middle-ear function was assessed using a Titan tympanometer. Speech audiometry was carried out using EAR-3A insert earphones and an Otometrics audiometer. SRT was obtained from both ears. The traditional clinical pure tone average (PTA) was calculated from the pure tone air conduction values at 500, 1,000 and 2,000 Hz. Additionally, the high-frequency PTA (HFPTA) was calculated from the averages of pure tone air conduction values at 1,000, 2,000, and 4,000 Hz. For clarity and analysis purposes, the pure tone average of the threshold values at 500, 1,000, and 2,000 Hz was labeled as the low-frequency pure tone average (LFPTA). A higher PTA indicates poorer hearing sensitivity. Noise-induced hearing loss typically results in a notched audiometric configuration at approximately 4,000 Hz. Adults with noise-induced hearing loss may experience this configuration bilaterally and typically have an asymmetric or unilateral hearing loss in terms of audiometric configuration. The older adults in the current sample showed bilateral, symmetrical hearing loss, indicating age-related hearing loss rather than noise-induced loss.

### 2.7 Statistical analysis

All statistical analyses were performed using GraphPad Prism version 10 and Statistical Package for the Social Sciences (SPSS) version 29. An independent (unpaired) samples t-test was used to determine group mean differences for subject characteristics, vascular hemodynamics, aortic stiffness, and auditory measurements between younger and older adults. Pearson product correlations were used to determine the relationship between aortic stiffness, AIxHR75 and aortic systolic BP (aSBP) and SRT, and LFPTA and HFPTA. Partial correlations were used to assess the relationship between aortic stiffness, AIxHR75, aSBP and SRT, and LFPTA and HFPTA in minimally adjusted models for markers of inflammation. Our sample size estimate (G*power 3.1) indicated that two-tailed correlational analyses required a combined sample size of n = 22 participants, which would be adequate to detect statistical significance with >80% power at α = 0.05 with a 0.56 effect size. All data are presented as mean ± SEM. A p-value < 0.05 was deemed statistically significant.

## 3 Results

A total of 22 participants completed the study. Participant characteristics, vascular hemodynamics, and audiological measurements of the participant population are presented in Table 1. Compared to younger adults, older adults had higher resting heart rate, cholesterol, LDL, aSBP, aortic PP, AIxHR75, and cfPWV (all, p < 0.05, Table 1). Bilateral hearing sensitivity was also lower in older adults than in younger adults, which was reflected as having a higher SRT and LFPTA and HFPTA in both the right and left ears (all, p < 0.05, Table 1). No differences were observed between markers of inflammation between younger and older adults (all, p > 0.05, Table 1).

**TABLE 1:** Subject characteristics.

Characteristics	Young (n = 11, 4 men/7 women)	Old (n = 11, 4 men/7 women)
Age, years	25.5 ± 2.1	65.8 ± 0.9*
Body mass, kg	83.1 ± 6.5	76.0 ± 5.5
HR_rest_, bpm	60.8 ± 2.4	70.1 ± 2.8*
BMI, kg/m^2^	27.7 ± 1.8	27.3 ± 1.3
BF, %	34.4 ± 3.3	37.7 ± 2.6
Cholesterol, mg/dL	166.0 ± 9.5	206.0 ± 9.9*
LDL, mg/dL	97.6 ± 9.6	127.0 ± 5.8*
HDL, mg/dL	51.4 ± 2.7	59.2 ± 4.5
Triglycerides, mg/dL	91.8 ± 12.6	101.0 ± 12.7
Glucose, mg/dL	90.7 ± 2.2	96.4 ± 2.8
Est CRF, mL•kg•min^−1^	36.4 ± 3.2	21.1 ± 2.2*
Cardiovascular hemodynamics		
^#^cfPWV, m/s	5.7 ± 0.3	7.0 ± 0.3*
AIx, %	11.8 ± 4.3	36.4 ± 3.9*
AIxHR75, %	4.3 ± 4.7	30.4 ± 3.8*
Brachial SBP, mmHg	105.0 ± 2.6	110.0 ± 2.9
Brachial PP, mmHg	38.0 ± 1.4	43.8 ± 2.1*
Aortic SBP, mmHg	104.0 ± 2.9	115.0 ± 3.1*
Aortic PP, mmHg	35.1 ± 2.1	36.8 ± 2.0
Auditory characteristics		
RE SRT, dB HL	4.1 ± 1.5	18.4 ± 1.6*
LE SRT, dB HL	4.1 ± 1.6	17.3 ± 1.6*
RE LFPTA, dB HL	4.9 ± 1.0	17.3 ± 2.1*
LE LFPTA, dB HL	3.9 ± 1.5	17.6 ± 1.9*
RE HFPTA, dB HL	4.4 ± 1.1	21.1 ± 2.6*
LE HFPTA, dB HL	4.6 ± 1.4	22.6 ± 2.9*
Inflammatory biomarkers		
Hs-CRP, g/L	1.72 ± 0.65	2.22 ± 0.59
^†^MMP-2, ng/mL	20.3 ± 1.6	18.7 ± 1.5
^†^Resistin, ng/mL	2.19 ± 0.22	1.64 ± 0.22
^†^Vaspin, pg/mL	425 ± 243	244 ± 80

^#^n = 21 (n = 10 for young/n = 11 for old).

^†^n = 10/group; AIx, augmentation index; AIxHR75, augmentation index normalized to heart rate 75; BMI, body mass index; BF, body fat; cfPWV, carotid–femoral pulse wave velocity; dB, decibel; HL, hearing level; HR, heart rate; HDL, high-density lipoprotein; HFPTA, high-frequency pure tone average; hs-CRP, high-sensitivity C-reactive protein; LE, left ear; LDL, low-density lipoprotein; LFPTA, low-frequency pure tone average; MMP-2, matrix metalloproteinase-2; PP, pulse pressure; RE, right ear; SRT, speech recognition threshold; SBP, systolic blood pressure. Data are shown as the mean ± SEM. *p < 0.05 vs. young.

Age was correlated with cfPWV (r = 0.61, p = 0.003), AIxHR75 (r = 0.73, p < 0.001), aSBP (r = 0.59, p = 0.004), RE (r = 0.82, p < 0.001) and LE (r = 0.81, p < 0.001) SRT, RE (r = 0.74, p < 0.001) and LE (r = 0.72, p < 0.001) LFPTA, and RE (r = 0.77, p < 0.001) and LE (r = 0.82, p < 0.001) HFPTA.

**FIGURE 1:**
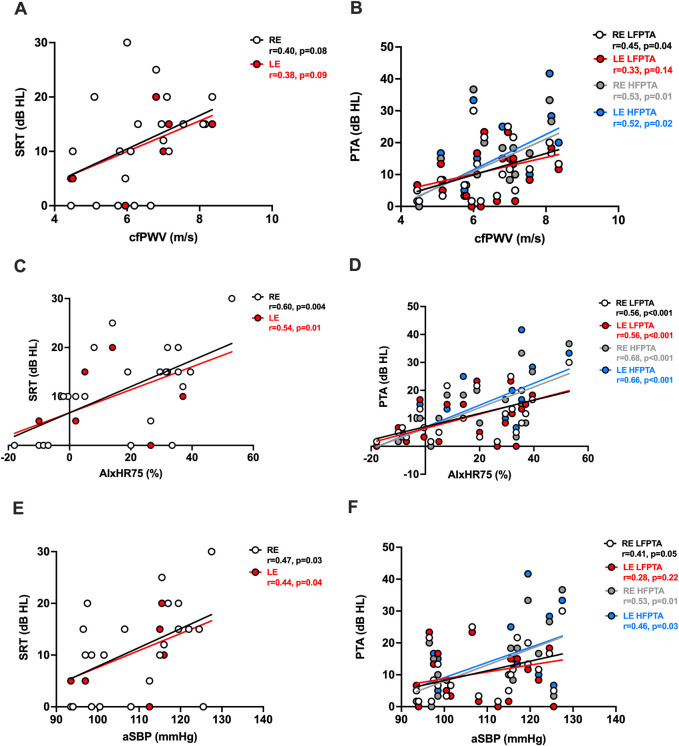
Higher arterial stiffness and aortic systolic blood pressure are associated with a higher speech recognition threshold and pure tone averages. Correlations between cfPWV and **(A)** SRT and **(B)** LFPTA and HFPTA; AIxHR75 and **(C)** SRT and **(D)** LFPTA and HFPTA; and aSBP and **(E)** SRT and **(F)** LFPTA and HFPTA. AIxHR75, augmentation index normalized to heart rate 75; aSBP, aortic systolic blood pressure; cfPWV, carotid–femoral pulse wave velocity; dB, decibel; HL, hearing level; HFPTA, high-frequency pure tone average; LE, left ear; LFPTA, low-frequency pure tone average; RE, right ear. cfPWV n = 21 (n = 10 for young/n = 11 for old); AIxHR75 and aSBP n = 22.

cfPWV was not correlated with SRT in either the RE (r = 0.40, p = 0.08) or LE (r = 0.38, p = 0.09) (both, Figure 1A). However, AIxHR75 and aSBP were both correlated with SRT in the RE (AIxHR75: r = 0.60, p = 0.004; aSBP: r = 0.47, p = 0.03) and LE (AIxHR75: r = 0.54, p = 0.01; aSBP: r = 0.44, p = 0.04) (all, Figures 1C,E). cfPWV was correlated with RE LFPTA (r = 0.45, p = 0.04) and both RE (r = 0.53, p = 0.01) and LE (r = 0.52, p = 0.02) HFPTA (all, Figure 1B) but not with LE LFPTA (r = 0.33, p = 0.14, Figure 1B). AIxHR75 and aSBP were correlated with RE (AIxHR75: r = 0.56, p < 0.01; aSBP: r = 0.41, p = 0.05) LFPTA and RE (AIxHR75: r = 0.68, p < 0.001; aSBP: r = 0.53, p = 0.01) and LE (AIxHR75: r = 0.66, p < 0.001; aSBP: r = 0.46, p = 0.03) HFPTA (all, Figures 1D,F). aSBP was not correlated with LE LFPTA (r = 0.28, p = 0.22, Figure 1F). There were no correlations between AIxHR75, cfPWV and aSBP, and hearing loss variables after adjusting for age (all, p > 0.05, Supplementary Table S1). Brachial systolic blood pressure was not related to SRT or PTA (both, p > 0.05, data not shown); however, brachial pulse pressure was correlated with RE (r = 0.49, p = 0.02) and LE (r = 0.55, p = 0.007) HFPTA.

Resistin tended to be correlated with AIxHR75 (r = 0.37, p = 0.10), LE LFPTA (r = −0.38, p = 0.09), and LE HFPTA (r = −0.44, p = 0.05); vaspin tended to be correlated with cfPWV (r = 0.35, p = 0.14); and MMP-2 tended to be correlated with LE HFPTA (r = −0.40, p = 0.08).

AIxHR75, cfPWV, and aSBP were independently correlated with hearing loss-related variables; however, no correlation was observed after independent adjustment for age (all, p > 0.05, Supplementary Table S1). The correlation between cfPWV and RE LFPTA and HFPTA in both ears did not remain after adjusting for MMP-2 or resistin (both, p > 0.05, Table 2). However, the relationship between AIxHR75 and SRT and LFPTA and HFPTA PTA in both ears remained after minimally adjusting for each inflammatory biomarker, namely, MMP-2, resistin, and vaspin (all, p < 0.05, Table 2). The relationship between aSBP and RE SRT and RE HFPTA remained (both, p < 0.05, Table 2) after adjusting for MMP-2, resistin, and vaspin. The relationship between aSBP and LE SRT and LE HFPTA also remained significant after adjusting for resistin (both, p < 0.05, Table 2). Additional inflammatory markers including CRP did not influence the relationship between cfPWV, AIxHR75, aSBP and SRT, or LFPTA and HFPTA (all, p > 0.05, data not shown).

**TABLE 2:** Correlations between aortic stiffness, AIxHR75, and aortic blood pressure and auditory characteristics adjusted for inflammatory biomarkers.

C	cfPWV, m/s				AIxHR75, %				aSBP, mmHg			
	Un-adjusted	MMP-2-adjusted	Resistin-adjusted	Vaspin-adjusted	Un-adjusted	MMP-2-adjusted	Resistin-adjusted	Vaspin-adjusted	Un-adjusted	MMP-2-adjusted	Resistin-adjusted	Vaspin-adjusted
Auditory variables	R	R	R	R	R	R	R	R	R	R	R	R
RE SRT, dB HL	0.40	0.41	0.41	0.41	0.60*	0.55*	0.52*	0.58*	0.47*	0.46*	0.51*	0.46*
LE SRT, dB HL	0.38	0.37	0.37	0.39	0.54*	0.47*	0.45*	0.51*	0.44*	0.42	0.48*	0.43
RE LFPTA, dB HL	0.45*	0.40	0.41	0.42	0.56*	0.50*	0.49*	0.52*	0.41*	0.37	0.41	0.39
LE LFPTA, dB HL	0.33	0.26	0.26	0.31	0.56*	0.49*	0.46*	0.52*	0.28	0.22	0.29	0.25
RE HFPTA, dB HL	0.53*	0.44	0.44	0.51*	0.68*	0.61*	0.62*	0.65*	0.53*	0.49*	0.57*	0.54*
LE HFPTA, dB HL	0.52*	0.41	0.41	0.47*	0.66*	0.58*	0.59*	0.63*	0.46*	0.39	0.51*	0.44

AIxHR75, augmentation index normalized to heart rate 75; aSBP, aortic systolic blood pressure; cfPWV, carotid–femoral pulse wave velocity; dB, decibel; HL, hearing level; LE, center ear; LFPTA, low-frequency pure tone average; HFPTA, high-frequency pure tone average; MMP-2, matrix metalloproteinase-2; RE, right ear; SRT, speech recognition threshold. *p < 0.05.

## 4 Discussion

This study provides evidence for the association of AIxHR75 and aSBP with age-related hearing loss. Specifically, our findings demonstrate that 1) AIxHR75 and aSBP but not cfPWV are associated with a higher SRT; 2) cfPWV, AIxHR75, and aSBP are associated with poorer hearing sensitivity (i.e., higher PTA) in both the low- and high-frequency domains; and 3) the relationship between both cfPWV and aSBP and LFPTA and HFPTA is influenced by age and MMP-2. Collectively, these preliminary findings demonstrate that higher aortic stiffness and aSBP are associated with lower hearing sensitivity with aging, which is influenced, in part, by MMP-2 in an apparently healthy, nonclinical population.

Aging is the primary risk factor contributing to the development of CVD and is associated with greater aortic stiffening and higher aortic blood pressure. Our findings demonstrate that AIxHR75 and aSBP, but not cfPWV, were positively correlated with a higher SRT, suggesting diminished hearing. SRT is used to measure the minimum intensity for speech processing, representing the minimum hearing level for speech at which individuals can recognize 50% of the speech material (Lee et al., 2024). Elevations in vascular hemodynamics compromise the structural and functional components of the microcirculation, leading to impaired blood flow to target organs such as the cochlea (Ungvari et al., 2021). Specifically, blood pressure has been shown to affect speed processing, working memory function, and short-term memory; however, the majority of studies examining the effects of blood pressure on SRT are limited to brachial blood pressure (Sánchez-Nieto et al., 2021). This is noteworthy as we extend these findings demonstrating that central (aortic) blood pressure and AIxHR75, but not cfPWV, are associated with a higher SRT in both the low- and high-frequency domains. These correlations did not remain significant after adjusting for age, suggesting age-dependency, thus supporting our primary hypothesis. Furthermore, the SRT test uses both auditory and language centers of the brain, which may explain, in part, the similarity between the performance of both the RE and LE and the correlation with cardiovascular parameters. As such, higher central blood pressure and AIxHR75 are predictors of mortality and thereby may serve as early indicators of hearing loss (Roman et al., 2007; Janner et al., 2013). Thus, these findings suggest that central blood pressure and AIxHR75 are important markers that may provide potential prognostic value with regard to auditory function and cognitive processing with aging.

Importantly, our findings demonstrate that cfPWV, the “gold standard” for assessing aortic stiffness, is associated with RE LFPTA and RE and LE HFPTA. Thus, these data support the notion that aortic stiffness plays a modulatory role with regard to hearing sensitivity in both the low- and high-frequency domains, which appears to be independent of ear dominance. Additionally, we demonstrate for the first time that AIxHR75 is associated with higher LFPTA and HFPTA and aSBP is associated with a greater HFPTA hearing threshold. Notably, cfPWV and AIxHR75 did not remain significantly correlated with hearing loss variables, supporting age as the key variable aligned with our hypothesis. Our results are supported by other studies, demonstrating that higher aortic pulse wave velocity is associated with higher PTA in older women, and not men, but only in the dominant hearing ear (Helzner et al., 2011). Brachial–ankle pulse wave velocity has also been shown to be associated with hearing loss with LFPTA in the dominant ear and with initial hearing levels in adults with idiopathic sudden sensorineural hearing loss (Chung et al., 2016; Tan et al., 2018). These data are noteworthy as aortic stiffness, augmentation index, and central blood pressure are not only predictors of increased CVD risk but are also associated with poorer cognitive performance and cognitive aging (Pase et al., 2013; Ou et al., 2020). As such, central blood pressure and augmentation index have been identified as sensitive indicators for the prediction of age-related cognitive performance that has not been predicted by brachial blood pressure (Pase et al., 2013). Thus, these data provide preliminary evidence for higher central arterial stiffness and aortic systolic blood pressure as contributors to age-related hearing loss.

Increases in aortic systolic blood pressure due to aortic stiffening with aging results in increased pulsatile pressure wave transmission to the microcirculation, which is reflective of higher augmentation index. Evidence from preclinical animal studies support this notion, demonstrating an impaired myogenic response of resistance arteries contributing to microvascular dysfunction as a potential mechanism underlying auditory dysfunction (Springo et al., 2015). Moreover, arteries distal to the large elastic arteries, such as the labyrinthine artery that supplies blood to the cochlea, are more susceptible to increased pulsatile energy, particularly when local microvascular arterial tone and resistance are low (Gratton et al., 1996; Shi, 2011). These data suggest that higher AIxHR75 and aSBP are important central vascular hemodynamics contributing to impaired hearing ability with aging.

Aortic stiffness, increases in central blood pressure, and hearing loss with aging are partly attributable to inflammatory processes (Verschuur et al., 2012; Verschuur et al., 2014; Jablonski et al., 2015; Zanoli et al., 2020; Pierce et al., 2022). A novel finding of this study was that the inflammatory molecules, MMP-2 and resistin, each attenuated the relationship between cfPWV and LFPTA and HFPTA, suggesting that these inflammatory biomolecules may be a link between arterial stiffness and hearing loss. A similar attenuation occurred for the relationship between aSBP and LE SRT and LE HFPTA after separately adjusting for MMP-2 and vaspin. Notably, current evidence demonstrates that higher circulating levels of MMP-2 and resistin are associated with aortic stiffness and hypertension and have been linked as a pathophysiological mechanism underlying presbycusis (Vlachopoulos et al., 2007; Norman et al., 2016; Wu et al., 2017; Zhang et al., 2017; Prado et al., 2021). As such, higher concentrations of MMP-2 and resistin are also associated with CVD and ischemic cerebrovascular disease-related events in older adults (Gencer et al., 2016; Wang et al., 2017). Therefore, it is reasonable to suggest that MMP-2 and resistin are important inflammatory biomarkers that may influence age-related hearing loss in adults without known CVD. Furthermore, while inflammation was not different at baseline between younger and older adults, this does not rule out low-grade inflammation contributing to hearing loss. Inflammation with aging has been shown to damage the inner structure of the cochlea and its sensory cells and promote blood vessel constriction, leading to reduced blood flow to the inner ear and ischemia contributing to the pathogenesis of hearing loss. Therefore, future studies are needed to further our understanding of the influence of inflammation on the relationship between cardiovascular dysfunction and hearing loss and aging (Eisenhut, 2019; Frosolini et al., 2022).

### 4.1 Strengths and limitations

The primary strengths of this pilot study were the use of cfPWV as the “gold-standard” measure of aortic stiffness and comprehensive evaluation of audiometric testing in a well-controlled and apparently healthy younger and older adult population without known CVD. However, this study is not without limitations. The generalizability of these findings may be limited due to 1) a small sample size of younger (n = 11) and older (n = 11) adults, 2) a normotensive and healthy vascular aging profile (age >50 years, cfPWV <7.6 m/s and brachial blood pressure <140/90 mmHg) in the older adult population, 3) the homogenous Caucasian population, and 4) the inability to establish temporal or casual effects from cross-sectional correlational analyses. Because of a strict inclusion and exclusion criteria of apparently healthy (i.e., free of known disease) adults to minimize the effect of confounding cardiovascular and metabolic risk factors on our primary outcomes, the extent to which aortic stiffness is associated with age-related hearing loss may not have been fully realized in this proof-of-concept pilot study. Future studies using a larger heterogeneous participant population in adults with a wider range of aortic stiffness, in those with and without hypertension, and in the presence and absence of comorbidities are needed to extend the findings of the current study. The influence of biological sex, blood pressure, arterial stiffness, and hearing loss with aging should be a primary emphasis in larger samples to understand how the relationship between measures of cardiovascular health differentially impacts auditory function in men and women. Furthermore, future studies assessing TNF-α and IL-6 as well-established inflammatory biomarkers mediating the vascular aging and auditory dysfunction associations are warranted to extend the current findings. Importantly, analyses regarding the temporal changes in arterial stiffness, blood pressure, inflammation, and sensory/auditory function are needed to better understand the impact of arterial dysfunction on auditory function with aging and elucidation of causal pathways. Incorporating cellular and experimental animal models are also necessary to more substantially identify the biological mechanisms by which inflammation may contribute to age-related increases in blood pressure and arterial stiffness leading to auditory dysfunction.

## 5 Conclusion

These findings provide novel evidence for the association of greater aortic stiffness and higher aortic systolic blood pressure with age-related hearing loss. Notably, these correlations were attenuated, in part, by both age and MMP-2 and resistin, highlighting both age and inflammation as potential contributing factors to aortic stiffness-related auditory dysfunction. Thus, these findings provide insights for aortic stiffness, aortic systolic blood pressure, and MMP-2 as clinically relevant therapeutic targets to prevent and/or mitigate presbycusis in adults without overt CVD.

## Data Availability

The original contributions presented in the study are included in the article/Supplementary Material, further inquiries can be directed to the corresponding author.
