# Supplementary material for: Age-related hearing loss in healthy older adults is associated with arterial stiffening and higher aortic systolic blood pressure: potential role of inflammation
Source: Front Aging. 2026 Feb 11;7:1642659. doi: 10.3389/fragi.2026.1642659 (PMC12932603; doi:10.3389/fragi.2026.1642659)
Supplement: Supplementary file 1 [file Table1.DOCX]

| **Supplemental Table 1: Correlations between aortic stiffness, AIxHR75 and aortic systolic blood pressure and auditory characteristics adjusted for age** | | | | | | |
| --- | --- | --- | --- | --- | --- | --- |
|  | **cfPWV, m/s** | | **AIxHR75, %** | | **aSBP, mmHg** | |
|  | **Un-**  **Adjusted** | **Age-Adjusted** | **Un-**  **Adjusted** | **Age-Adjusted** | **Un-**  **Adjusted** | **Age-Adjusted** |
|  | **R** | **R** | **R** | **R** | **R** | **R** |
| RE SRT, dB HL | 0.40 | -0.27 | 0.60***** | -0.01 | 0.47***** | -0.03 |
| LE SRT, dB HL | 0.38 | -0.29 | 0.54***** | -0.12 | 0.44***** | -0.08 |
| RE LFPTA, dB HL | 0.45***** | -0.01 | 0.56***** | 0.05 | 0.41***** | -0.04 |
| LE LFPTA, dB HL | 0.33 | -0.20 | 0.56***** | 0.09 | 0.28 | -0.25 |
| RE HFPTA, dB HL | 0.53***** | 0.11 | 0.68***** | 0.26 | 0.53***** | 0.16 |
| LE HFPTA, dB HL | 0.52***** | 0.13 | 0.66***** | 0.26 | 0.46***** | 0.05 |
| AIxHR75, augmentation index normalized to heart rate 75; aSBP, aortic systolic blood pressure; cfPWV, carotid-femoral pulse wave velocity; dB, decibel; HL, hearing level; LE, left ear; LFPTA, low-frequency pure tone average; HFPTA, high frequency pure tone average; RE, right ear; SRT, speech recognition threshold. *****p<0.05. | | | | | | |
